# Exploring the trend of age-standardized mortality rates from cardiovascular disease in Malaysia: a joinpoint analysis (2010–2021)

**DOI:** 10.1186/s12889-024-19103-7

**Published:** 2024-09-16

**Authors:** Wan Shakira Rodzlan Hasani, Kamarul Imran Musa, Kueh Yee Cheng, Sarat Chandra Dass

**Affiliations:** 1 Department of Community Medicine, School of Medical Sciences, Universiti Sains Malaysia, Kubang Kerian, Kelantan, 16150 Malaysia; 2 Biostatistics and Research Methodology Unit, School of Medical Sciences, Universiti Sains Malaysia, Kubang Kerian, Kelantan, 16150 Malaysia; 3 School of Mathematical and Computer Sciences, Heriot-Watt University Malaysia, Putrajaya, 62200 Malaysia

**Keywords:** CVD mortality, ASMR, Joinpoint analysis, Trend, COVID-19 pandemic

## Abstract

**Introduction:**

Cardiovascular disease (CVD) is a major health concern worldwide, particularly in low- and middle-income countries. The COVID-19 pandemic that emerged in late 2019 may have had an impact on the trend of CVD mortality. This study aimed to investigate the trend and changes in CVD mortality rates in Malaysia, using age-standardized mortality rates (ASMR) from 2010 to 2021.

**Methods:**

The Malaysian population and mortality data from 2010 to 2021 were obtained from the Department of Statistics Malaysia (DOSM). ASMRs from CVD per 100,000 population were calculated based on the World Health Organization (2000–2025) standard population using the direct method. The ASMRs were computed based on sex, age groups (including premature mortality age, 30–69 years), and CVD types. The annual percent change (APC) and average annual percent change (AAPC) of the ASMR with corresponding 95% confidence intervals (95% CI) were estimated from joinpoint regression model using the Joinpoint Regression Program, Version 4.9.1.0.

**Results:**

Throughout the study period (2010–2021), ASMRs for CVD exhibited an increase from 93.1 to 147.0 per 100,000, with an AAPC of 3.6% (95% CI: 2.1 to 5.2). The substantial increase was observed between 2015 and 2018 (APC 12.6%, 95% CI: 5.4%, 20.3%), with significant changes in both sexes, and age groups 50–69, 70 years and over, and 30–69 (premature mortality age). Notably, the ASMR trend remained consistently high in the premature mortality age group across other age groups, with males experiencing higher rates than females. No significant changes were detected before or after the COVID-19 pandemic (between 2019 and 2021), except for females who died from IHD (10.3% increase) and those aged 0–4 (25.2% decrease).

**Conclusion:**

Overall, our analysis highlights the persistently high burden of CVD mortality in Malaysia, particularly among the premature mortality age group. These findings underscore the importance of continued efforts to address CVD risk factors and implement effective prevention and management strategies. Further research is needed to fully understand the impact of the COVID-19 pandemic on CVD mortality rates and to inform targeted interventions to reduce the burden of CVD in Malaysia.

## Background

Cardiovascular disease (CVD) is the leading cause of death worldwide, responsible for approximately one-third of all deaths globally [1, 2]. Low- and middle-income countries (LMICs) bear a disproportionate burden of CVD, accounting for 32% of all CVD deaths [3]. Despite declining age-standardized mortality rates (ASMR) in high-income regions, most LMICs have not experienced similar reductions [4, 5]. Instead, there has been an increase in premature mortality (deaths that occur at a younger age than expected) from CVD in LMICs [6]. Recognizing this alarming trend, there is a global commitment to reducing premature CVDs by 25% by the year 2025 [7]. While significant progress has been made in preventing and treating CVD, there are notable disparities among different subpopulations, and the trend for CVD mortality has been inconsistent globally [5, 8–12]. Malaysia, classified as an LMIC, also faces a significant burden of CVD, particularly ischemic heart disease (IHD) and stroke [13].

Changes in CVD mortality trends can be attributed to several factors. Adverse conditions or behaviours related to modifiable risk factors such as diabetes, obesity, lack of physical activity, hypertension, an unhealthy diet, smoking, and excessive alcohol consumption are associated with an increase in CVD mortality trends [4, 14–16]. Without reducing these risk factors, it is predicted that almost 23.6 million people will die from CVDs by 2030 [17]. Other factors, including an aging population [18], environmental factors [19], and changes in healthcare access and quality [15], may contribute to these disparities in the trend for CVD mortality.

In addition to these factors, the COVID-19 pandemic, which began in late December 2019 [20] has had a significant impact on the healthcare system and may have affected the trend for CVD mortality. For example, the pandemic resulted in excess all-cause mortality in the USA [21] and in a multi-country study using data from 74 countries worldwide [22]. In Poland, in-hospital mortality for acute heart failure increased [23], whereas in Sweden, CVD mortality, particularly from IHD and myocardial infarction, decreased substantially during 2020 [24]. Several countries reported a decrease in hospitalizations and percutaneous coronary interventions during the COVID-19 pandemic, likely leading to a reduction in recorded mortality from cardiac events [25–27]. Lockdowns and prioritization of COVID-19 care may have indirectly affected diseases like CVDs that require a functional healthcare system [28, 29]. Thus, analysing mortality trends allows for understanding the indirect impacts of the pandemic on health outcomes, identifying gaps in healthcare delivery, and developing strategies to mitigate these impacts.

Joinpoint analysis is a statistical method that is commonly used to detect changes in trends or patterns in data over time, and it has been used in many previous studies to investigate the burden of various diseases, including CVD [30–33]. Investigating the trend for CVD mortality within a country is crucial, particularly in LMICs like Malaysia. However, to date, the trend and any change in trend of ASMR from CVD in Malaysia have not been investigated over the past decade, including the impact of COVID-19 on CVD mortality rates. Using joinpoint analysis to analyse Malaysian data will provide valuable insights into the temporal patterns of CVD mortality occurrence in Malaysia and help inform policy and intervention strategies to reduce the burden of CVD in the country. Moreover, it will contribute to the literature on CVD mortality trends and change point analysis, particularly in LMICs, and provide a basis for future studies on CVD mortality in the region. Therefore, this study aims to explore the change in trend of ASMR from CVD in Malaysia from 2010 to 2021. Specifically, we aim to identify any changes in trends according to sex, major CVD types, and age group, including premature mortality. Furthermore, we aim to assess any changes in the CVD mortality trend within each subgroup before and after the COVID-19 pandemic.

## Methods

### Source of data

Data on CVD mortality and Malaysian population (census and inter-census years) were obtained from the Department of Statistics Malaysia (DOSM) for the period 2010–2021. Malaysian law mandates that all deaths be registered with the National Registration Department (NRD), which issues death certificates [34]. Deaths in Malaysia are categorized into medically certified deaths, which occur in health facilities and are determined by medical officers based on symptoms and examination, and non-medically certified deaths, which occur outside health facilities. While death registration quality is an issue in many countries [35]. Malaysia stands out as one of the few Asian countries with a functioning vital registration system. Analysing trends from 1995 to 2010, medically certified deaths increased over time, while non-medically certified deaths remained stable [36]. In 1995, non-medically certified deaths were 55%, surpassing medically certified deaths at 45% [36]. In 2021, DOSM reported an improvement, with medically certified deaths at 70.0% and non-medically certified deaths at 30.0% [37]. In Malaysia, death certificates document a clear sequence of events leading to death. The cause of death listed on the lowermost line of the sequence, which initiated the train of events leading to death, is defined as the underlying cause of death. All other causes listed on the lines between the underlying cause (on the lowermost line) and the immediate cause (on the topmost line) are referred to as antecedent causes of death [38]. DOSM is responsible for cleaning and classifying cause-of-death information for all medically certified deaths obtained from the NRD, coding them based on the 10th International Classification of Diseases (ICD-10) [39]. This process is carried out by specialized coders at DOSM. To ensure the study’s quality and enhance the accuracy of cause-of-death information, only medically certified deaths for CVD as indicated by ICD-10 (code I01-I99) were used for this analysis. The unknown cause of death and missing information on age and sex were excluded.

### Statistical analysis

We estimated ASMRs per 100,000 population for all medically certified deaths from CVD. The World (WHO 2000–2025) Standard population [40] was used as the reference population to calculate ASMR, based on the direct method of age-standardization. The use of a standard population is important to enable comparability between relevant years and rates from other countries. To calculate the ASMR using direct standardization, we first aggregated the number of CVD deaths into 5-year age intervals (0–4, 5–9, 10–14, 15–19, 20–24, 25–29, 30–34, 35–39, 40–44, 45–49, 50–54, 55–59, 60–64, 65–69, 70–74, 75–79 and 80+) for each year of study. Next, we calculated the age-specific mortality rates by dividing the number of deaths in a specific age group by the number of people in that age group and then multiplying the result by 100,000. Finally, the ASMR was calculated by multiplying the age-specific death rate by the weight of that age group in the standard population [40]. This direct standardization method was applied to calculate the overall ASMR, and stratified by gender (male and female), CVD type (IHD and stroke), and selected specific age ranges (e.g., 0–1, 30–49, 50–59 years). To explore the trend for premature CVD mortality, we also selected a specific age group of 30–69 years, in accordance with the WHO definition of premature mortality [41]. The descriptive statistics of ASMR and trend plots were analysed using R software.

Joinpoint regression analysis was utilized to detect the significant changes in annual ASMR for CVD mortality from 2010 to 2021 according to sex, age groups and CVD types. A joinpoint regression model was employed to estimate the magnitude of change in the trend of CVD mortality rates over time. This model allows us to identify points in time, where significant changes occur in the trend. Additionally, the model provides a confidence measure around these estimated changes. We used the Joinpoint Regression Program, Version 4.9.1.0 for this analysis [42]. The Joinpoint program fits a series of straight lines to the ASMRs on a log scale and detects the best fitting points, called ‘joinpoints’ [43]. This program selects the final model using two methods; the Monte Carlo permutation tests and the Bayesian Information Criterion (BIC) [43]. The analysis starts with the minimum number of joinpoints (e.g., zero joinpoints, which is a straight line), and tests whether one or more joinpoints are significant and must be added to the model. Based on the recommendation of the Joinpoint program, a maximum of two joinpoints can be selected for the given 12 data points [42]. Permutation tests determine the number of joinpoints by comparing different hypotheses until the final number is reached. Starting with ka = minimum number of joinpoint and kb = maximum number of joinpoint, each test compares the null hypothesis H0: number of joinpoints = ka against the alternative Ha: number of joinpoints = kb, where ka < kb. If the null hypothesis is rejected, ka is increased by 1; otherwise, kb is decreased by 1. This process continues until ka = kb, and the final value is the selected number of joinpoints. Then, models with this number of joinpoints are compared using BIC, and the model with the minimum BIC value is selected as the optimal model [44].

To describe changes in CVD mortality rates, the annual percent change (APC) of the ASMR between the trend-change point and the average annual percent change (AAPC) in the whole period studied was calculated with corresponding 95% confidence intervals (95% CI). We applied the log transformation to calculate the APC. This approach assumes that CVD mortality rates change at a constant percentage relative to the rate of the previous year, and the log transformation allows for linear changes on a logarithmic scale. To derive the APC for a given data series, the following regression model is employed [45];

$$\text{log}\left({R}_{y}\right)={b}_{0}+{b}_{1}y$$

where $\text{log}\left({R}_{y}\right)$ is the natural log of the rate in year $y$. ${b}_{0}$is the intercept, representing the value of the natural log of the rate when $y=0,$ and ​${b}_{1}$ is the slope, indicating the rate of change in the natural log of the rate per year.

The APC from year $y$ to year $y$ + 1 is derived from this formula

$$\left[\frac{{R}_{y+1}-{R}_{y}}{{R}_{y}}\right]\times \text{ }100$$

The formula is further transformed to represent an annual percent change;.

$$APC =\frac{\left\{{e}^{{b}_{0}+{b}_{1}\left(y+1\right)}-{e}^{{b}_{0}+{b}_{1}\left(y\right)}\right\}}{{e}^{{b}_{0}+{b}_{1}\left(y\right)}}\times \text{ }100$$

$$=\left({e}^{{b}_{1}}-1\right)\times \text{ }100$$

The AAPC was calculated to summarize the trend over a specific fixed interval (2010–2021), allowing for the use of a single number to describe the APCs over multiple years. The AAPC is computed by taking a weighted average of the APCs from the joinpoint model, with the weights determined by the length of each APC interval. The AAPC over any fixed interval is calculated by taking a weighted average of the slope coefficients of the underlying joinpoint regression model, where the weights correspond to the length of each segment within the interval [46].

APC_i_ = {exp(b_i_) − 1} x 100.

where b_i_ is the slope coefficient for the i^th^ segment (within the desired range of years).

In the calculation, the weighted average of slope coefficients is further transformed to represent an average annual percent change [46];

$$AAPC=\left\{\text{exp}\left(\frac{\sum {w}_{i}{b}_{i}}{\sum {w}_{i}}\right)-1\right\}\times \text{ }100$$

where w_i_ is the length of each segment within that range.

The Joinpoint Regression Program performs a series of hypothesis tests. These tests compare the null hypothesis (the assumption of no trend or no joinpoints) against alternative hypotheses with different numbers of joinpoints. A small *p*-value indicates strong evidence against the null hypothesis, suggesting significant trends or joinpoints. The trend was considered to be significantly increasing (positive change) or decreasing (negative change) when the *p*-value was below 0.05 (*p* < 0.05).

## Results

Table 1 shows the demographic structure of the Malaysian population and the age standardized mortality rate from CVD. The Malaysian population is systematically increasing for the period 2010–2021 from 28.6 million to 32.6 million [47]. During the observed period, 1.9 million deaths (from all causes) were recorded in Malaysia, of which 318,268 deaths (16.6%) were caused by CVD.

**Table 1:** Population structure and age standardised mortality rate from CVD in Malaysia from 2010 to 2021

Year	All CVD							Main CVD type			
	Total			Male		Female		IHD		Stroke	
	Population Malaysia (‘000)	*n*	ASMR^a^	*n*	ASMR^a^	*n*	ASMR^a^	*n*	ASMR^a^	*n*	ASMR^a^
2010	28,588.60	18,842	93.1	11,974	116.0	6868	69.6	9371	46.4	4763	24.0
2011	29,062.00	19,895	95.1	12,663	119.1	7232	70.5	10,004	47.8	5112	24.9
2012	29,510.00	19,985	91.7	12,784	115.8	7201	67.4	10,091	46.3	5220	24.4
2013	30,213.70	20,033	88.1	12,862	111.4	7171	64.4	10,169	44.9	5156	22.9
2014	30,708.50	20,986	88.7	13,535	112.5	7451	64.5	10,432	44.0	5474	23.6
2015	31,186.10	21,976	89.1	14,125	112.7	7851	65.1	11,018	44.5	5678	23.4
2016	31,633.50	22,802	89.0	14,821	114.1	7981	63.7	11,310	43.9	5876	23.4
2017	32,022.60	27,342	104.2	17,504	132.0	9838	76.3	13,503	51.1	6878	26.8
2018	32,382.30	36,334	135.8	22,751	168.3	13,583	103.2	18,267	67.6	9154	34.9
2019	32,523.00	32,748	117.7	20,828	148.7	11,920	87.0	16,325	58.3	8691	31.8
2020	32,447.40	35,841	131.1	22,733	163.0	13,108	98.5	18,515	67.1	9101	34.1
2021	32,576.30	41,484	147.0	25,950	180.7	15,534	112.7	21,485	75.5	10,181	36.7

Abbreviations: CVD: Cardiovascular disease (ICD-10 code: I01-I99); IHD: Ischemic heart disease (ICD-10 code: I20-I25); Stroke: including all cerebrovascular disease (ICD-10 code: I60-I69); n is number of deaths from cardiovascular disease

^a^The World Health Organization world standard population was used for age standardization, and the age standardized rate was calculated per 100,000 population

### Age standardised mortality rate

**Fig. 1:**
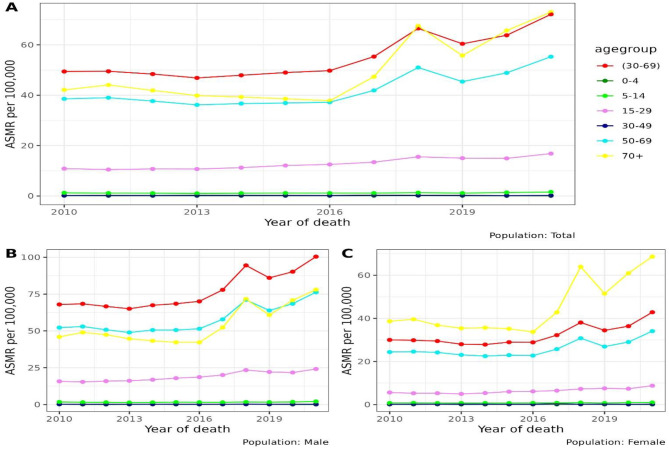
The ASMR trend from CVD in Malaysia from 2010 to 2021; (**A**) Overall population by age group; (**B**) Male by age group and (**C**) Female by age group

The ASMR ranged from 88.1 to 147.0 per 100,000 population (Table 1). The overall ASMR showed an increase trend from 93.1 to 147.0 deaths per 100,000 population from 2010 to 2021, but with small variation between years. During the study period, the rates were almost twice as high in men as in women. The trend shown increases in both sexes and the main CVD types (IHD and stroke). Although all of the rates increased over the period of study, the pattern of increase varied across age groups. Figure 1 shows that the ASMR trend in males was highest among those aged 30–69 years (defined as premature mortality), whereas in females, the highest rate was observed in those over 70 years of age. We detected that the trend for both sexes was rising and peaking in 2018, particularly among the premature mortality age group and those over 50. Meanwhile, the mortality trend among adolescents and children below the age of 15 for both sexes has almost plateaued over the years.

### Joinpoint regression model

Table 2 shows the joinpoint regression analysis of ASMR for total CVD and the model for both sexes stratified by age group and CVD type. The jointpoint model demonstrated a statistically significant increase in ASMR for CVD, with an AAPC of 3.6% (95% CI: 2.1, 5.2) during the entire study period (2010 to 2021). However, when we looked at a specific segment, the trend was slightly decreasing between 2010 and 2015, with an APC of -2.2% (95% CI: -3.6, -0.7), and a substantial increase between 2015 and 2018, with an APC of 12.6% (95% CI: 5.4, 20.3). Between 2018 and 2021, the rate increased slightly (APC: 5.0%, 95% CI: 1.6%, 8.6%), and no significant changes were detected before or after the COVID-19 pandemic (2019–2021). These changes in ASMRs exhibited similar patterns in both men and women, with large increments occurring between 2015 and 2018.

**Fig. 2:**
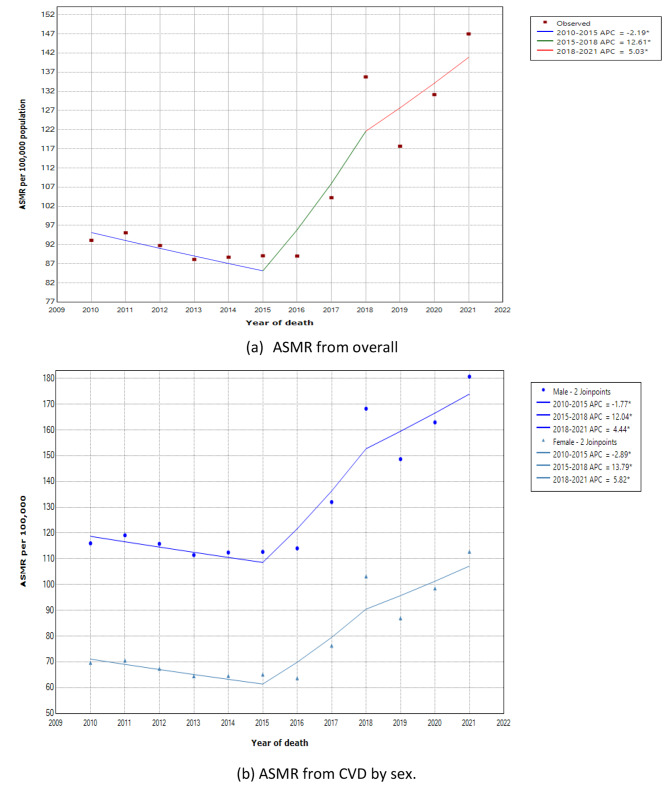
Joinpoint regression model of age standardised mortality rate (ASMR) per 100,000 from CVD in Malaysia, 2010–2021 for overall CVD (**a**) and by sex (**b**)

**Fig. 3:**
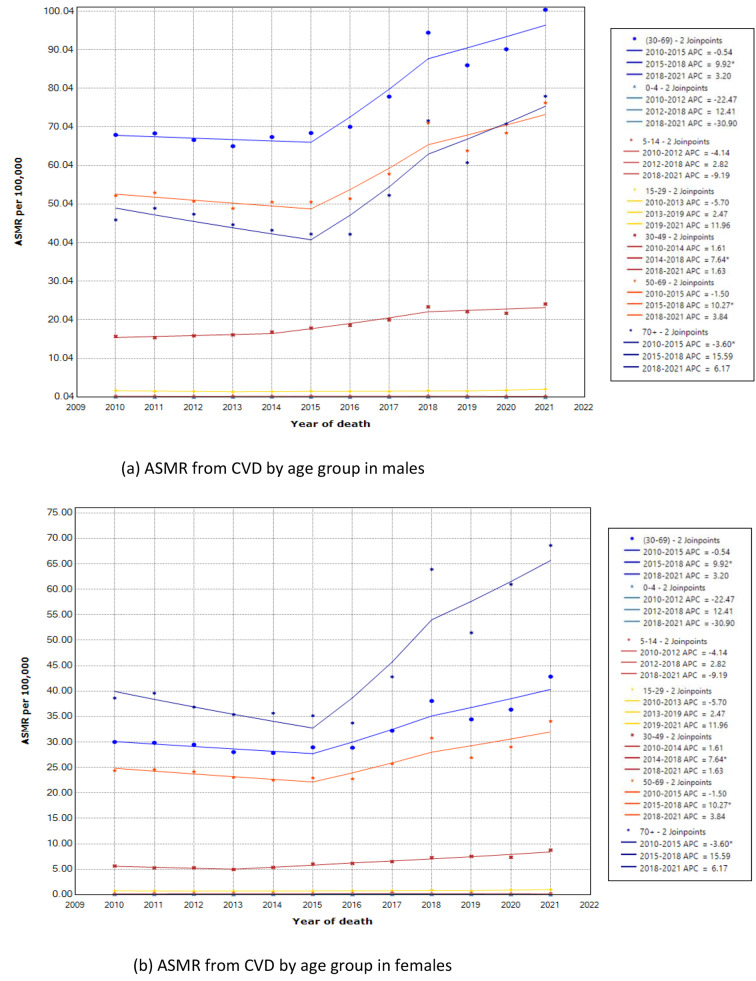
Joinpoint regression model of age standardised mortality rate (ASMR) per 100,000 from CVD in Malaysia, 2010-2021by age group in male (**a**) and female (**b**)

The analysis of trends by age strata (Table 2) demonstrated the large increase between 2015 and 2018 was contributed by the age groups 50–59, ≥ 70 years, and premature mortality group, with APCs of 9.5%, 16.7%, and 9.3%, respectively. Meanwhile, the reduction in trend between 2010 and 2015 was notable among females aged 50–69 (reduced by -2.3%) and both male and female aged ≥ 70 age groups (reduced by -3.6% in females and − 3.9% in males). Interestingly, among premature CVD mortality (age 30–69), the trend was systematically increasing throughout the study period, where the reduction slopes between 2010 and 2015 were not statistically significant. In addition, the AAPC during the observed study (2010–2021) only significantly increased among the aged group over 30 years. Meanwhile, the mortality rate in the age group below 30 years was nearly constant, and the trend in this subgroup was not statistically significant, except for those aged 0–4 years, who showed a significant decline in trend (reduced by 25.5%) from 2019 to 2021. Figures 2 and 3 present the joinpoint model plots illustrating the overall ASMR trend from CVD, as well as the trends by sex and age group.

In terms of CVD subtype, both IHD and stroke showed an upward trend between 2010 and 2021, with AAPC rates of 3.9% and 3.4%, respectively (Table 2). Although females reported lower ASMR from IHD and stroke than males, females had a greater increase in trend for IHD and stroke. Specifically, the AAPC for females was 4.5% for IHD and 3.6% for stroke, whereas for males it was 3.8% for IHD and 3.3% for stroke.

**Table 2:** Join point regression model of age standardized mortality rate from CVD in Malaysia, 2010–2021

		Total			Male			Female		
Cohort		Time period	APC (95% CI)	*p* value*	Time period	APC (95% CI)	*p* value	Time period	APC (95% CI)	*p* value
Total		2010–2015	-2.2 (-3.6, -0.7)	**0.014**	2010–2015	-1.8 (-3.3, -0.3)	**0.032**	2010–2015	-2.9 (-4.3, -1.4)	**0.005**
		2015–2018	12.6 (5.4, 20.3)	**0.008**	2015–2018	12.0 (4.6, 20.0)	**0.010**	2015–2018	13.8 (6.5, 21.6)	**0.006**
		2018–2021	5.0 (1.6, 8.6)	**0.015**	2018–2021	4.4 (0.9, 8.1)	**0.024**	2018–2021	5.8 (2.4, 9.4)	**0.009**
		AAPC	3.6 (2.1, 5.2)	**< 0.001**	AAPC	3.5 (1.9, 5.2)	**< 0.001**	AAPC	3.8 (2.3, 5.4)	**< 0.001**
Age group (years)										
	0–4	2010–2015	-3.3 (-13.3, 7.8)	0.439	2010–2012	-22.5 (-63.8, 65.9)	0.406	2010–2013	-4.5 (-17.3, 10.2)	0.422
		2015–2018	15.6 (-28.9, 87.9)	0.455	2012–2018	12.4 (-5.2, 33.3)	0.129	2013–2019	2.8 (-3.6, 9.6)	0.296
		2018–2021	-25.5 (-41.6, -5.0)	**0.028**	2018–2021	-30.9 (-52.8, 1.1)	0.054	2019–2021	-25.2 (-43.9, -0.4)	**0.048**
		AAPC	-5.5 (-15.3, 5.6)	0.318	AAPC	-8.0 (-19.9, 5.7)	0.239	AAPC	-4.9 (-9.7, 0.2)	0.059
	5–14	2010–2014	2.4 (-13.1, 20.7)	0.707	2010–2012	-4.1 (-40.4, 54.1)	0.817	2010–2012	12.6 (-52.1, 164.6)	0.719
		2014–2017	3.4 (-38.5, 74.0)	0.865	2012–2018	2.8 (-7.5, 14.3)	0.508	2012–2017	1.8 (-22.3, 33.4)	0.860
		2017–2021	-5.9 (-20.2, 10.9)	0.361	2018–2021	-9.2 (-28.4, 15.2)	0.323	2017–2021	-5.2 (-27.7, 24.2)	0.610
		AAPC	-0.4 (-11.4, 11.9)	0.943	AAPC	-1.9 (-10.0, 7.0)	0.670	AAPC	1.0 (-13.6, 18.1)	0.896
	15–29	2010–2013	-4.6 (-11.7, 3.1)	0.166	2010–2013	-5.7 (-15.4, 5.1)	0.208	2010–2013	-2.1 (-10.8, 7.4)	0.554
		2013–2019	2.5 (-1.0, 6.1)	0.121	2013–2019	2.5 (-2.4, 7.6)	0.236	2013–2019	2.3 (-1.9, 6.6)	0.204
		2019–2021	12.6 (-3.6, 31.5)	0.101	2019–2021	12.0 (-9.9, 39.1)	0.222	2019–2021	12.7 (-6.4, 35.7)	0.149
		AAPC	2.2 (-0.6, 5.2)	0.126	AAPC	1.8 (-2.1, 5.9)	0.377	AAPC	2.9 (-0.6, 6.4)	0.102
	30–49	2010–2013	-0.6 (-6.2, 5.3)	0.774	2010–2014	1.6 (-2.0, 5.3)	0.285	2010–2013	-3.5 (-12.3, 6.3)	0.365
		2013–2018	6.8 (3.0, 10.8)	**0.007**	2014–2018	7.6 (1.7, 13.9)	**0.023**	2013–2016	7.6 (-11.3, 30.4)	0.352
		2018–2021	3.4 (-2.4, 9.5)	0.187	2018–2021	1.6 (-4.0, 7.6)	0.473	2016–2021	6.1 (1.6, 10.8)	**0.019**
		AAPC	3.8 (1.8, 5.9)	**< 0.001**	AAPC	3.8 (1.7, 5.9)	**< 0.001**	AAPC	3.8 (-0.6, 8.4)	0.095
	50–69	2010–2015	-1.8(-3.4, -0.1)	**0.040**	2010–2015	-1.5 (-3.4, 0.5)	0.100	2010–2015	-2.3 (-4.3, -0.2)	**0.037**
		2015–2018	9.5 (1.6, 18.0)	**0.028**	2015–2018	10.3 (1.0, 20.4)	**0.037**	2015–2018	8.1 (-1.4, 18.6)	0.078
		2018–2021	4.1 (0.3, 8.0)	**0.040**	2018–2021	3.8 (-0.6, 8.5)	0.076	2018–2021	4.5 (-0.2, 9.5)	0.056
		AAPC	2.8 (1.1, 4.5)	**0.001**	AAPC	3.1 (1.0, 5.1)	**0.003**	AAPC	2.3 (0.2, 4.5)	**0.031**
	70+	2010–2015	-3.7 (-6.2, -1.2)	**0.015**	2010–2015	-3.6 (-6.7, -0.4)	**0.035**	2010–2015	-3.9 (-6.1, -1.7)	**0.008**
		2015–2018	16.7 (4.1, 30.8)	**0.020**	2015–2018	15.6 (-0.1, 33.6)	0.050	2015–2018	18.2 (6.8, 30.8)	**0.010**
		2018–2021	6.5 (0.6, 12.7)	**0.038**	2018–2021	6.2 (-1.2, 14.1)	0.083	2018–2021	6.7 (1.5, 12.3)	**0.023**
		AAPC	4.3 (1.6, 7.0)	**0.001**	AAPC	4.0 (0.6, 7.5)	**0.020**	AAPC	4.6 (2.2, 7.1)	**< 0.001**
	30–69	2010–2015	-0.9 (-2.6, 0.8)	0.206	2010–2015	-0.5 (-2.3, 1.3)	0.457	2010–2015	-1.6 (-3.8, 0.6)	0.109
	(Premature	2015–2018	9.3 (1.3, 18.0)	**0.031**	2015–2018	9.9 (1.3, 19.2)	**0.032**	2015–2018	8.2 (-2.0, 19.5)	0.092
	Mortality) **	2018–2021	3.8 (-0.1, 7.8)	0.054	2018–2021	3.2 (-0.9, 7.5)	0.098	2018–2021	4.7 (-0.4, 10.1)	0.061
		AAPC	3.1 (1.3, 4.9)	**0.001**	AAPC	3.2 (1.4, 5.2)	**0.001**	AAPC	2.7 (0.4, 5.0)	**0.021**
Main CVD type										
	IHD	2010–2015	-2.4 (-3.9, -0.8)	**0.014**	2010–2015	-1.9 (-3.6, -0.1)	**0.047**	2010–2016	-1.7 (-2.7, -0.7)	**0.010**
		2015–2018	12.1 (4.5. 20.4)	**0.011**	2015–2018	12.1 (3.3, 21.7)	**0.018**	2016–2019	14.1 (7.4, 21.3)	**0.004**
		2018–2021	6.6 (2.9, 10.4)	**0.007**	2018–2021	5.4 (1.1, 9.8)	**0.024**	2019–2021	10.3 (3.8, 17.2)	**0.011**
		AAPC	3.9 (2.2. 5.5)	**< 0.001**	AAPC	3.8 (1.9, 5.7)	**< 0.001**	AAPC	4.5 (3.0, 6.1)	**< 0.001**
	Stroke	2010–2015	-1.9 (-4.1, 0.3)	0.074	2010–2015	-1.5 (-4.4, 1.5)	0.244	2010–2015	-2.5 (-4.5, -0.4)	**0.030**
		2015–2018	12.3 (1.5, 24.2)	**0.033**	2015–2018	10.8 (-3.1, 26.7)	0.100	2015–2018	14.2 (3.9, 25.5)	**0.017**
		2018–2021	4.1 (-1.0, 9.5)	0.089	2018–2021	4.3 (-2.4, 11.5)	0.154	2018–2021	3.8 (-0.9, 8.9)	0.090
		AAPC	3.4 (1.1, 5.8)	**0.004**	AAPC	3.3 (0.3, 6.5)	**0.034**	AAPC	3.6 (1.4, 5.8)	**0.001**

Joinpoint regression model applied. The significance of the joint points determined using the Monte Carlo permutation test. The maximum number of joinpoints was set at 2 (ICD-10 code: I60-I69)

* *p* value for hypothesis tests that test the null hypothesis of ka (minimum number of joinpoints) against the alternative hypothesis of kb (maximum number of joinpoints), where ka and kb change for each hypothesis test (ka < kb)

**Aged 30–69 is defined as premature mortality for this study

During the period from 2019 to 2021, which encompasses the pre- and post-COVID-19 pandemic eras, our joinpoint analysis revealed significant changes in CVD mortality rates among females. Specifically, there was a notable increase in the mortality rate due to IHD among females, with an APC of 10.3%. Additionally, there was a significant decrease in mortality among female children aged 0–4, with an APC of -25.2%. However, it’s important to note that significant changes among males were detected between 2018 and 2021, which falls outside the defined pre- and post-COVID-19 pandemic period of 2019–2021.

## Discussion

In this study, we conducted joinpoint analysis to identify and measure temporal patterns of ASMR on CVD mortality in Malaysia from 2010 to 2021. These analytical methods proved useful in understanding the underlying drivers of the observed trends and their implications for policies and interventions. Our findings indicated an overall increase in ASMR from CVD over the study period (AAPC of 3.6%) with a large increment occurring between 2015 and 2018 (APC of 12.6%). Interestingly, we detected a slight decrease in the trend between 2010 and 2015 (APC of -2.2%). This descending trend in CVD mortality aligns with other reports worldwide. For example, the findings from the GBD Study 2017 reported that nearly all countries, including developing countries and regions like Southeast Asia, experienced a significant declining trend in age-standardized CVD mortality rates from 1990 to 2017 [48]. A study by Khan et al. revealed that the global mortality trend of IHD decreased slowly but progressively from 1990 to 2017 [49]. They suggested that this reduction may be partly due to increasing global awareness of lifestyle factors. Another study showed that the ASMR of stroke decreased sharply by 33.4% over the same period [50].

However, several epidemiological studies have reported a global trend of age-standardized CVD mortality rates either slightly declining or increasing in most LMICs, while high-income regions have experienced a significant reduction [4, 18]. For example, a study in Central Asia (comprising LMICs) found that CVD mortality trends have risen over the past two decades [51]. The researchers attributed this rise to factors such as inadequate preventive care, low awareness of disease signs and symptoms, reduced physical activity, elevated blood pressure, and insufficient utilization of healthcare services.

Although a slight reduction occurred before 2015, our joinpoint model suggests that the ASMR for CVD in Malaysia increased throughout the study period (2010–2021). The observed increase in ASMR from CVD in this country may be attributed to a combination of factors, including urbanization, changes in lifestyle and dietary habits, and a shift towards more sedentary lifestyles [52–54]. These factors have contributed to unhealthy, high-calorie diets, leading to the development of metabolic disorders like obesity and diabetes, which are well-known risk factors for CVD. Over the past decade, Malaysia has experienced a significant increase in the prevalence of several metabolic disorders, including obesity, diabetes, hypertension, and hypercholesterolemia. According to the National Health and Morbidity Survey (NHMS), the prevalence of obesity among adults in Malaysia has risen from 14.0% in 2006 [55] to 17.7% in 2019 [56], while the prevalence of diabetes has increased from 11.6% in 2006 [55] to 18.3% in 2019 [56]. The survey also showed that the prevalence of hypertension and hypercholesterolemia among adults in Malaysia has been increasing from 32.2% and 20.6% in 2006 [55] to 43.5% and 47.7% in 2019 [56], respectively. These trends are concerning, as these metabolic disorders are significant risk factors for CVD, which could contribute to the observed increase in ASMR from CVD in Malaysia. Additionally, Malaysia is experiencing a demographic transition due to an increasing aged population ≥ 60 years and increased life expectancy [57]. Population aging is becoming the most important driver of the CVD epidemic [58]. On the other hand, increased access to healthcare and better medical treatment for CVD [59, 60] may have also led to higher reported CVD mortality rates. Firstly, increased access to healthcare can lead to better detection and diagnosis of CVD cases, including previously undiagnosed or asymptomatic cases. As a result, more individuals with CVD are identified and included in mortality statistics, thus contributing to higher reported mortality rates. Secondly, better medical treatment for CVD can prolong the lives of individuals with the condition. While this is beneficial for improving individual health outcomes, it can also increase the pool of individuals living with CVD, who are at risk of dying from CVD-related complications in the long term. This, in turn, can lead to higher reported mortality rates.

While there has been an overall upward trend in CVD mortality rates, our joinpoint regression analysis only identified significant changes pre- and post-COVID-19 outbreak (2019–2021) among females who died from IHD and females aged 0–4 years, with no significant changes detected in other subgroups during this period (2019–2021). It is important to note that the impact of COVID-19 on CVD mortality rates during the early phases of the pandemic is inconsistent, with some countries reporting a decrease in CVD mortality [61–63] and others having observed an increase or excess mortality [64–66]. While our data is limited to one-year post-COVID-19 pandemic (2021), the study by Jayaraj et al. [67] on all-cause mortalities in Malaysia between January 2016 and September 2021 also utilized similar post-COVID data points up to 2021. Their results show a reduction in all-cause mortality in 2020, especially during the first Movement Control Order, followed by a significant increase between July and September 2021. This pattern supports our findings and might explain why the APC did not show significant changes between 2019–2021 in our study, despite an overall significant increase in the APC between 2018–2021. Additionally, the use of monthly data points with all causes of death in their study might have allowed for more precise detection of changes in trends during the COVID-19 pandemic compared to our study, which used yearly data points with specific causes of death.

On the other hand, our study highlights the persistent burden of premature CVD mortality (age 30–69) in Malaysia, which is in line with the trend of increasing global premature CVD mortality observed in LMICs [1]. Furthermore, there are notable sex disparities, with higher rates of premature CVD mortality among males compared to females. This finding is consistent with the results of Zhang et al. (2021) [68], who reported a 35.6% higher overall premature CVD mortality rate among men than women, based on global data from the WHO Global Health Estimates (GHE). Other studies [69–71] have also highlighted sex disparities related to premature CVD mortality.

In addition, our study revealed that while males had a higher overall ASMR than females, the magnitude of increase during the entire study period (2010–2021) was greater among females (AAPC 3.8% in females versus 3.5% in males). Notably, females aged over 70 years exhibited the highest ASMR over time compared to other age groups. These findings are consistent with the observations of Roth et al. [1], who reported a rapid increase in the proportion of CVD-related deaths among women after the age of 70, surpassing that among men. However, Roth et al. found that this trend is driven predominantly by stroke mortality, whereas our findings show that IHD had a higher increment among Malaysian females. These findings highlight the importance of targeted interventions and policies to address sex-specific CVD types and age-related disparities in ASMR from CVD mortality in Malaysia.

### Study limitations

The study had some limitations that should be taken into consideration when interpreting the results. Firstly, the use of yearly data points and the lack of post-COVID-19 outbreak observation may have contributed to the insignificant findings of changes in the overall CVD mortality trend and most of the subgroup during the pre- and post-COVID-19 outbreak period (2019–2021). Therefore, the true burden of the effect of COVID-19 on CVD deaths should be interpreted with caution. Despite this limitation, our intention to examine the potential impact of the early COVID-19 pandemic on CVD mortality remains relevant, as evidenced by the significant APC detected during the pre- and post-COVID-19 period (2019–2021) in certain groups (e.g., females with IHD). Additionally, the study was limited by the variables available in the death registry, which did not include information on other modifiable risk factors such as diabetes, hypertension, and alcohol use, as well as important sociodemographic factors such as ethnic group, regional area, income level, and employment status. These variables may have been important to adjust or stratify in the joinpoint regression analysis. Furthermore, the study may not have represented the total CVD deaths in Malaysia, as it relied only on medically certified deaths. However, the use of the most complete and accurate data on CVD death, with ICD-10 coding conducted by specialist coders from DOSM and validated by independently certified coders, is a strength of the study.

## Conclusion

In conclusion, this study highlights the increasing trend in ASMR from CVD in Malaysia, with a substantial increase observed between 2015 and 2018. The findings also underscore the ongoing burden of premature CVD mortality in the country, particularly among males. Efforts to address CVD risk factors and implement effective prevention and management strategies should be continued, including public health campaigns to raise awareness about healthy lifestyle behaviours, enhancing access to affordable and quality healthcare services, strengthening primary healthcare systems, investing in community-based interventions, and promoting multi-sectoral collaboration. Further research is warranted to explore the specific impact of the COVID-19 pandemic on CVD mortality rates in Malaysia and to guide the development of targeted interventions to mitigate its effects.

## Data Availability

The mortality data used in this study were obtained from a restricted source (Department of Statistics Malaysia). The population structure in Malaysia is available online (https://pqi.stats.gov.my). We confirm that all methods were carried out in accordance with the relevant guidelines and regulations.

## Abbreviations

AAPC: the average annual percent change.
APC: the annual percent change.
IHD: Ischemic heart disease (ICD-10 code: I20-I25).
Stroke: including all cerebrovascular disease.
